# Thread-Embedded-in-PDMS Wearable Strain Sensor for Real-Time Monitoring of Human Joint Motion

**DOI:** 10.3390/mi14122250

**Published:** 2023-12-17

**Authors:** Mingpeng Yang, Yongquan Liu, Wenjing Yang, Jia Liu

**Affiliations:** 1 School of Automation, Nanjing University of Information Science and Technology, 219 Ningliu Road, Nanjing 210044, China; mpyang@nuist.edu.cn (M.Y.); 202212490609@nuist.edu.cn (Y.L.); 2 Jiangsu Collaborative Innovation Centre on Atmospheric Environment and Equipment Technology, Nanjing University of Information Science and Technology, 219 Ningliu Road, Nanjing 210044, China; 3 School of Atmospheric and Remote Sensing, Wuxi University, 333 Xishan Avenue, Wuxi 214105, China; ywj030114@outlook.com

**Keywords:** wearable strain sensor, good linearity, human joint motion, PBT thread, PDMS

## Abstract

Real-time monitoring of human joint motion holds paramount importance in assessing joint health status, preventing and treating joint diseases, and evaluating physical flexibility and coordination. However, traditional strain sensors face limitations in meeting the substantial strain requirements associated with human joint motion. Recently, there has been considerable attention directed towards flexible strain sensors prepared using pliable substrates combined with silk and cotton fabrics. Nonetheless, these sensors exhibit insufficient linearity across the entire measurement range, thereby compromising the predictability of real joint motion based on the output signal. This paper introduced a flexible strain sensor designed to address this issue by offering an enhanced range and high linearity. Specifically, the core wire of the strain sensor was produced by coating a polybutylene terephthalate thread with conductive carbon ink integrated with carbon nanotubes, encapsulated in a thin layer of polydimethylsiloxane in an “S” configuration. The proposed strain sensor maintained excellent linearity within its strain range of 60%, along with advantages such as rapid response speed and robust durability. On-trial tests further affirmed the sensor’s capability to effectively monitor the motion of human joints.

## 1. Introduction

Wearable sensors have garnered significant attention due to their ability to monitor real-time human biophysical and biochemical information [1,2,3,4,5,6]. The real-time monitoring of human joint motion poses a crucial and challenging task in human motion tracking, particularly as traditional strain sensors struggle to meet the demanding requirement for large strain measurements during joint motion [7,8,9].

Flexible strain sensors offer a solution by being adaptable to mechanical actuators or the outer surface of the human skin, enabling the acquisition of real-time strain information. In contrast to conventional strain sensors, flexible strain sensors employ pliable substrate materials that facilitate substantial deformations, significantly expanding the range of strain measurements. This adaptability allows them to accommodate diverse joint movements and postures, showcasing distinctive advantages in domains such as robotic motion control and human motion monitoring [10,11,12].

Flexible strain sensors commonly use flexible materials as substrates and utilize impedance (resistance, capacitance, inductance) changes or the piezoelectric effect to construct strain–impedance or strain–voltage conversion, achieving real-time measurement of strain [13,14]. Among them, resistance-type flexible strain sensors have attracted much attention due to their high sensitivity, ease of integration, and fast response [15,16,17]. Usually, resistance-type flexible strain sensors are composed of individual flexible core wires or can be composed of flexible core wires combined with flexible substrates (such as cloth or polymers) [18,19].

The construction of the core wire constitutes a pivotal phase in the fabrication of flexible strain sensors [20,21]. Traditionally, sensors are crafted by subjecting the fabric to carbonization, resulting in a core wire with inherently high conductivity. However, the carbonization process is intricate, demanding high temperatures and extended carbonization times, culminating in a less flexible core wire that is prone to fractures. This necessitates secure embedding within a pliable substrate material, and strain sensors fashioned with carbonized fabric wires typically exhibit a limited linear strain range. An alternative approach involves preparing a flexible sensor core wire by coating a conductive material onto a flexible thread. For instance, Yang et al. [22] utilized carbon nanotubes as the conductive material and employed an immersion method to create strain sensors, showcasing a substantial strain range. Nevertheless, this method fails to maintain linearity across the entire strain range. In another instance, Wang et al. [23] developed graphene-silk fabric strain sensors for human motion detection, exhibiting a high resistance change rate with increasing strain; however, their performance was confined to a linear strain range of 0–10%. Despite noteworthy advancements in conductive fabric-based strain sensors, the majority of reported sensors struggle to simultaneously achieve both excellent linearity and a large strain range.

In this investigation, a blend of conductive carbon ink (CCI) and carbon nanotubes (CNTs) was meticulously coated onto a polybutylene terephthalate (PBT) fluffy wire, renowned for its exceptional elasticity, to craft the core wire of the strain sensor. Subsequently, this core wire was encapsulated in a delicate layer of polydimethylsiloxane (PDMS), configured in a specific shape to prevent detachment of the conductive layer, enhance the sensor’s durability, and improve wearer comfort. Through meticulous adjustments to the core wire configuration, the sensor maintained exemplary linearity while achieving a commendable strain capacity. The evaluation encompassed scrutiny of hysteresis characteristics, response speed, bending attributes, and the overall service life of the sensor. In the concluding phases, the flexible strain sensor was applied to measure the movements of various human body joints, including the wrist, elbow, finger, and knee. This comprehensive assessment further validated the sensor’s practical performance.

## 2. Experiment

### 2.1. Materials and Equipment

Polybutylene terephthalate (PBT) bandages were procured from Shenzhen Aijia Medical Equipment Co., Ltd. (Shenzhen, China), and multi-walled carbon nanotubes (MWCNTs) (>90%, internal diameter 5–10 nm) were purchased from Shanghai Maclean Biochemical Technology Co., Ltd. (Shanghai, China). Polydimethylsiloxane (PDMS) elastomer (Sylgard 184) was purchased from Dow Corning (Midland, MI, USA). The drying oven was obtained from Dongguan Lixian Instrument Technology Co., Ltd. (Dongguan, China).

Maintaining stringent environmental conditions, the laboratory exercised precise control over temperature, regulating within the range of 22 °C to 24 °C and ensuring a relative humidity level of 55% to 60%.

### 2.2. Fabrication of the Sensors

**Figure 1:**
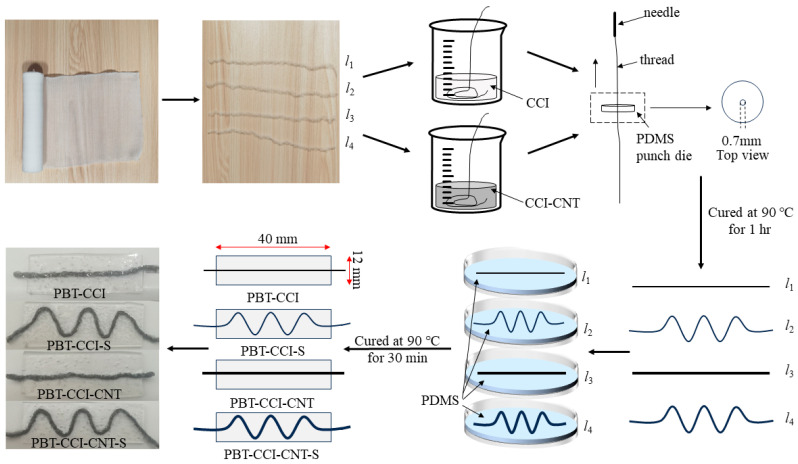
Fabrication process of the strain sensors.

As illustrated in Figure 1, four distinct types of strain sensors were manufactured employing various core wires: PBT thread coated with conductive carbon ink (PBT-CCI) and encapsulated in PDMS in a linear configuration; PBT thread coated with conductive carbon ink and encapsulated in PDMS in an “S” shape (PBT-CCI-S); PBT thread coated with a mixture of conductive carbon ink and multi-walled carbon nanotubes and encapsulated in PDMS in a linear configuration (PBT-CCI-CNT); and PBT thread coated with a mixture of conductive carbon ink and multi-walled carbon nanotubes and encapsulated in PDMS in an “S” shape (PBT-CCI-CNT-S).

Prior to sensor fabrication, a punch die was crafted to facilitate thread straightening during the manufacturing process. The PDMS and curing agent were meticulously mixed in a 10:1 ratio, with bubble removal carried out in a vacuum chamber. Subsequently, the mixture was poured into a mold with a 5 mm height and cured in a 90 °C oven for 30 min. A puncher was employed to drill a hole with a 0.7 mm diameter in the PDMS block.

Four PBT fluffy threads were extracted from the PBT bandage (as depicted in Figure 1), and their tensile limit exceeded 60% of the initial length. The conductive carbon ink was poured into a beaker, and two PBT fluffy threads were fully immersed and stirred in the conductive carbon ink. To ensure uniformity in the conductive layer of the PBT fluffy threads, a sewing needle (diameter 1 mm) was used to draw the threads through the fabricated punch die. The perforated PBT fluffy threads were then dried in an oven for 30 min at 90 °C. Placed in both linear and “S” shape configurations, the two fluffy threads were secured in a dish. Simultaneously, PDMS and a curing agent were mixed at a 20:1 ratio, with bubble removal, and the resulting mixture was poured into the dish to achieve a liquid level of 2 mm. Following a 30 min curing process in an oven at 90 °C, the PBT-CCI and PBT-CCI-S strain sensors were successfully fabricated.

A total of 2 g conductive carbon ink and 100 mg of carbon nanotubes were mixed in a beaker and thoroughly stirred. Subsequently, two additional PBT fluffy threads were directly immersed in the mixture and thoroughly coated. Using a sewing needle with a diameter of 1 mm, the PBT fluffy threads were guided through a PDMS punch die with a thickness of 5 mm. The perforated PBT fluffy threads were then dried in a 90 °C oven for 30 min. Following the drying process, the PBT fluffy threads were respectively arranged into linear and “S” shapes, as illustrated in Figure 1, and securely fixed in the mold. Concurrently, a mixture of PDMS and a curing agent in a 20:1 ratio, with careful removal of bubbles, was poured into the mold, ensuring a PDMS liquid height of 2 mm. The assembly was cured in a 90 °C oven for 30 min, resulting in the fabrication of the PBT-CCI-CNT and PBT-CCI-CNT-S strain sensors.

### 2.3. Measurement Circuit and Apparatus

**Figure 2:**
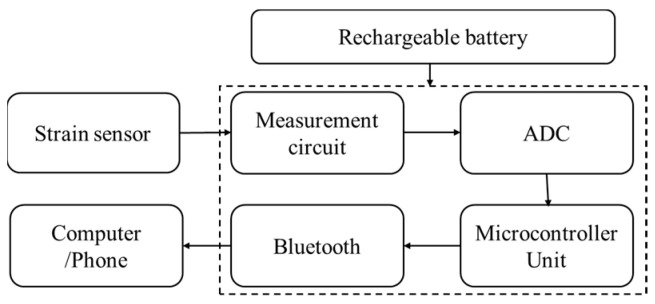
Signal processing and transmission workflow for the strain sensor.

In our study, we devised a data acquisition module to collect output data from the sensor. The schematic diagram is depicted in Figure 2, and detailed circuit design drawings are provided in Figures S1 and S2 (Supplementary Materials). The resistance measurement circuit captures the strain resistance of the sensor, converts it into voltage, undergoes conditioning, and subsequently outputs it to the ADC module. This analog signal is then transformed into a digital signal, transmitted to the microcontroller and, facilitated by Bluetooth technology, enables real-time data transmission and storage within the terminal.

**Figure 3:**
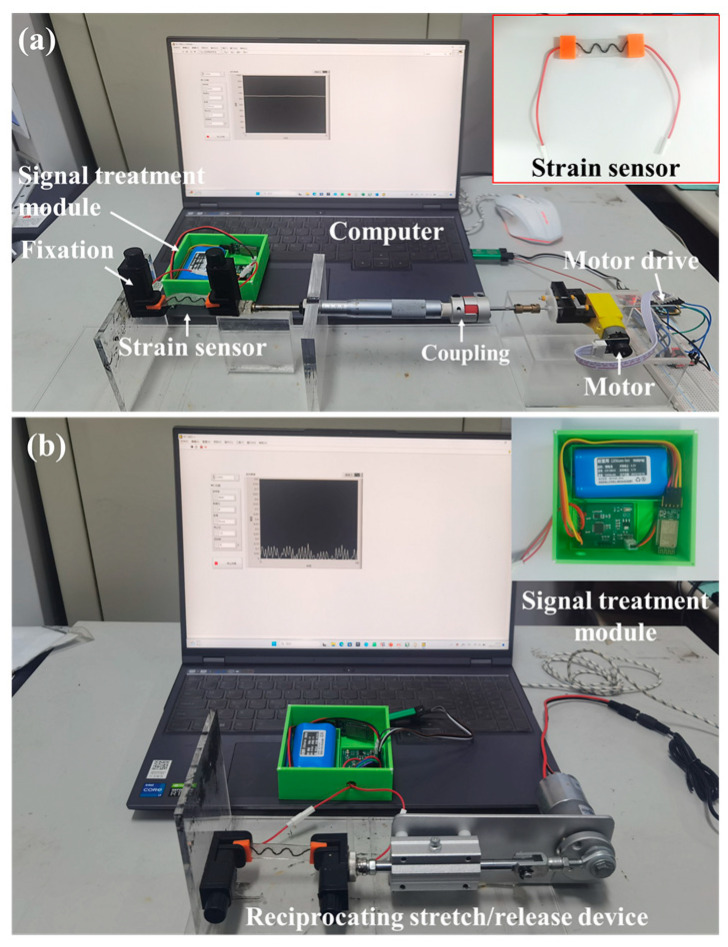
Apparatus for evaluating the physical characteristics of strain sensors. (**a**) Apparatus for low-speed stretch/release of the sensor; (**b**) apparatus for high-speed stretch/release of the sensor.

Two distinct apparatuses were devised to systematically evaluate the physical characteristics of the strain sensor. The configuration illustrated in Figure 3a was employed to measure the strain-resistance profile of the sensor, facilitating the assessment of sensitivity and linearity. Concurrently, this apparatus was utilized to gauge the hysteresis characteristics of the sensor. By employing a coupling mechanism, a screw micrometer, and miniature plain bearings, the apparatus translated the rotational movement of the electrodes into linear motion at the screw micrometer’s front end, enabling precise low-speed stretch/release measurements of the sensor. The device depicted in Figure 3b was employed for cyclic testing and durability cycle testing of the sensor. Leveraging a linkage mechanism, this setup seamlessly converted motor rotation into the linear motion of the push rod, allowing for expedited stretch/release measurements of the sensor. These two distinct setups comprehensively addressed various facets of the strain sensor’s performance, providing comprehensive insights into sensitivity, linearity, hysteresis, and durability under cyclic testing conditions.

### 2.4. Microscopic Characterization and Initial Resistance Measurement of the Sensor Core Wires

Through Scanning Electron Microscopy (SEM), we conducted an examination of the surface morphology of the three types of core wires: PBT, PBT-CCI, and PBT-CCI-CNT. This investigative step allowed us to systematically evaluate the effective adhesion of conductive ink and carbon nanotubes to the PBT fibers.

Concurrently, we performed resistance measurements on various types of core wires to assess the impact of conductive ink and carbon nanotube coatings on the resistance of PBT. Utilizing the clamping device illustrated in Figure 3, the three types of core wires were precisely straightened to their initial lengths. Subsequently, a commercial multimeter was employed to measure the resistance of each wire. Three measurements were taken for each type of core wire, and the average value was recorded for comprehensive analysis.

### 2.5. Performance Evaluation of the Strain Sensors

In the pursuit of accurate measurements of the strain-resistance output performance for the four strain sensors prepared in Section 2.2—specifically, PBT-CCI, PBT-CCI-S, PBT-CCI-CNT, and PBT-CCI-CNT-S—and the subsequent assessment of their sensitivity and linearity to identify the optimum strain sensor, we employed the low-speed stretching apparatus illustrated in Figure 3a. The sensors were gradually stretched at a rate of 0.6 mm/s until reaching 60% deformation (consistent with the previously established strain gauge stretching deformation limit of 60%).

Real-time resistance data were meticulously recorded using the custom-designed resistance measurement circuit described earlier. The recorded data were seamlessly transmitted and stored on a computer via Bluetooth technology. Experimental conditions were maintained at a temperature of 24.1 °C and a humidity level of 50%.

### 2.6. Comprehensive Electromechanical Performance Evaluation of PBT-CCI-CNT-S Strain Sensor

After thorough evaluation, it was determined that the sensor configuration of PBT-CCI-CNT-S was particularly well-suited for detecting human joint movements. Consequently, a comprehensive assessment of this sensor’s performance was conducted. Throughout all tests, the environmental conditions were maintained at a temperature of 24.1 °C and a humidity level of 50%.

To investigate the impact of stretching and releasing rates on the strain detection performance of the sensor, cyclic stretching and releasing tests were performed on the strain pieces using the apparatus depicted in Figure 3b. The stretching deformation of the strain pieces was set at 25%, and tests were carried out at rates of 3 mm/s, 6 mm/s, and 9 mm/s, each for 5 cycles.

In order to scrutinize the hysteresis characteristics of the sensor, slow stretching and releasing tests were conducted on the strain pieces using the setup illustrated in Figure 3a. The stretching and releasing rate of the strain pieces was set to 0.6 mm/min. The pieces were stretched to 10%, 25%, 40%, and 60%, followed by a release at the same rate to obtain the hysteresis curve of the strain pieces.

To validate the response rate of the sensor, performance tests were carried out on the strain pieces using the apparatus shown in Figure 3b. The stretching deformation of the strain pieces was set at 25%, and the stretching test was conducted at a rate of 6 mm/s.

To examine the response characteristics of the sensor at different strain levels, performance tests were performed on the strain pieces using the apparatus depicted in Figure 3b. The stretching rate of the strain pieces was set to 3 mm/s, and 5 cycles of stretching and releasing tests were conducted at strain levels of 10%, 20%, 30%, and 40%.

To assess the impact of bending behavior on the strain detection performance of the sensor, strain-resistance response tests were conducted under conditions where one end of the strain piece was fixed, and the other end was simply supported. Additionally, tests were conducted under conditions where both ends were fixed. The downward displacement at the midpoint of the strain piece was set to 0.5 mm, 1 mm, 1.5 mm, and 2 mm, with bending tests conducted accordingly.

To verify the cyclic stability of the sensor, durability tests were conducted on the strain pieces using the setup shown in Figure 3b. The stretching rate of the strain pieces was set to 3 mm/s, and 1000 cycles of stretching and releasing were conducted at a 25% strain level.

### 2.7. On-Trial Tests

To further validate the sensor’s practical performance, we conducted wearable tests on the human wrist, elbow, finger, and leg joints (knee region). A volunteer with a height of 182 cm, weight of 75 kg, and leg length of 98 cm participated in the tests. The PBT-CCI-CNT-S strain sensor was worn on various joint locations of the volunteer, and the obtained signals were wirelessly transmitted to a terminal in real time, recording the test data.

The strain sensor was placed on the upper, left, and right sides of the volunteer’s wrist joint. The wrist was actively moved, and the sensor’s output data were recorded in real time during each flexion, approaching approximately a 90-degree position. This testing procedure was repeated for a duration of 20 s at each wrist position to ensure comprehensive data collection.

Similarly, the sensor was positioned on the upper, left, and right sides of the volunteer’s elbow joint. The elbow was flexed to around 60 degrees, and the sensor’s output data were recorded in real time during each movement. This testing process, lasting 20 s at each elbow position, was conducted to gather comprehensive and coherent data.

Concurrently, the sensor was also tested on the finger. However, due to the impracticality of attaching the sensor to the side of the finger joint, it was affixed on the upper side, based on experience. Initially, cyclic bending effects were tested at a 60-degree flexion. Subsequently, the response was measured at finger flexions of 30 degrees, 60 degrees, and 90 degrees. Lastly, the strain response was measured during gripping/releasing a small object. 

Lastly, the sensor was worn on the front, left side, and right side of the volunteer’s leg joint. The volunteer performed tests with small steps (approximately 40 cm/step), medium steps (approximately 70 cm/step), and large steps (approximately 90 cm/step). For each distinct position, all three step lengths were tested for 20 seconds each, totaling 60 seconds of observation and recording of the sensor readings.

## 3. Results and Discussion

### 3.1. Microscopic Morphology and the Resistance of the Core Wires

**Figure 4:**
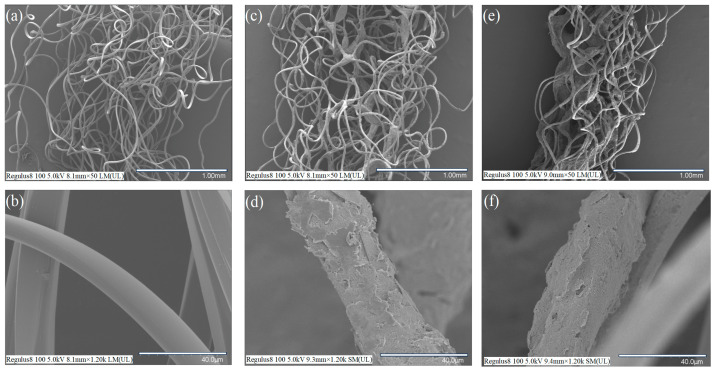
Surface morphology of the strain sensors: (**a**) scanning electron microscope (SEM) image of the pristine PBT and (**b**) its fiber surface; (**c**) SEM image of PBT coated with conductive carbon ink and (**d**) its fiber surface; (**e**) SEM image of PBT coated with a mixture of conductive carbon ink and carbon nanotubes and (**f**) its fiber surface.

The scanning electron microscope (SEM) images in Figure 4a,b provide a comprehensive view of the PBT fluffy thread and the surface of its fibers. The surface exhibits remarkable smoothness, without any visual discernible particles. Upon modification with conductive carbon ink, as depicted in Figure 4c,d, the ink uniformly and successfully coats the surface of the PBT thread. Moving on to Figure 4e,f, the mixture of conductive carbon ink and carbon nanotube particles is shown to effectively cover both the PBT fabric and the surfaces of individual fibers, creating a dense network of conductive particles within the fabric. This dense particle network predominantly forms within the continuous wave fabric and along individual fibers. The combined particles of conductive carbon ink and carbon nanotubes adhere securely to the surface of the conductive fabric and its individual fibers, facilitated by mechanical interlocking and hydrogen bonding. Clear observations from the scanning electron microscope confirm the presence of densely packed carbon nanoparticles on both the surface and within the fibers. It is inferred that carbon nanotube particles act as bridges, improving the connectivity of the conductive carbon ink. This enhancement significantly increases the number of conductive pathways and improves the conductivity of the fibers.

The inherent insulating nature of the pure PBT wire undergoes a transformative shift to conductivity upon the application of conductive carbon ink (CCI) or carbon nanotubes (CNTs). The resistance characteristics of the three distinct core wires (PBT-CCI, PBT-CCI-CNT) were meticulously assessed, yielding resistance values of 73 ± 5 kΩ/cm and 151 ± 20 kΩ/cm, respectively. This analysis clearly demonstrates that the application of CCI effectively converts the PBT wire from an insulating medium to a conductive one. Furthermore, the introduction of CNT contributes to an increment in the resistivity of the core wire, elucidating a nuanced understanding of the electrical properties associated with these modifications.

### 3.2. Performance Testing and Comparison of Four Strain Sensors

**Figure 5:**
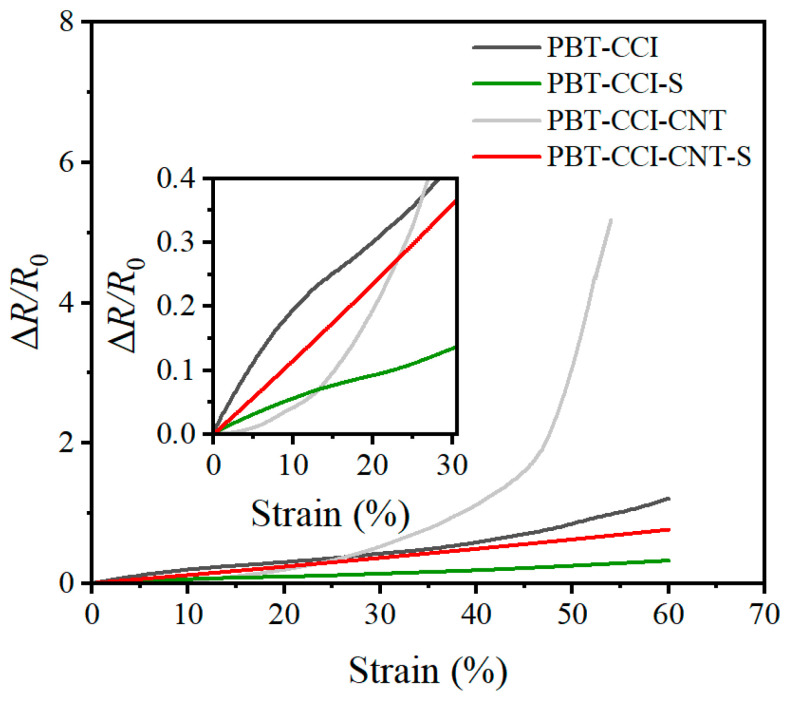
Relative resistance variation of four types of strain sensors at 60% tensile strain.

As delineated in Section 2, four distinct strain sensors were devised. Comprehensive performance assessments were conducted on these sensors to evaluate their sensitivity and linearity. Employing the apparatus illustrated in Figure 3a, the strain sensors underwent slow elongation, reaching 60% at a rate of 0.6 mm/min, as depicted in Figure 3. The resulting output relationship between resistance alteration and strain is depicted in Figure 5.

Notably, PBT-CCI and PBT-CCI-CNT exhibited comparatively higher sensitivity when contrasted with PBT-CCI-S and PBT-CCI-CNT-S strain sensors. However, they manifested stronger nonlinearity within the 10–30% and 40–60% strain ranges. Conversely, PBT-CCI-S and PBT-CCI-CNT-S, although not showcasing remarkable sensitivity, demonstrated outstanding linearity across the entire range. The linear core wires of PBT-CCI and PBT-CCI-CNT resulted in nearly identical strain rates with the deformation of the PDMS elastomer during sensor elongation. In contrast, the S-shaped core wires of PBT-CCI-S and PBT-CCI-CNT-S introduced more strain tolerance, as the wire’s deformation rate was less than that of PDMS during stretching, providing these structures with a greater deformation margin. Therefore, S-shaped core wires (PBT-CCI-S and PBT-CCI-CNT-S) offered superior linearity. The linear ranges and the corresponding linearity for each sensor are summarized in Table 1.

Figure 5 further illustrates that PBT-CCI-CNT and PBT-CCI-CNT-S strain sensors exhibited superior sensitivity compared to PBT-CCI and PBT-CCI-S sensors. The key distinction between the two groups lies in the inclusion of CNT in the core wire. The heightened sensitivity is attributed to the addition of carbon nanotubes in both PBT-CCI-CNT and PBT-CCI-CNT-S, with the hollow structure of CNT creating more void spaces in the conductive layer of PBT fluffy thread. This structure had increased resistivity compared to the solid CCI conductive layer, while the core wire was rendered more responsive to structural deformations, thereby enhancing sensitivity.

In summary, the incorporation of CNT enhances sensitivity, making PBT-CCI-CNT and PBT-CCI-CNT-S preferable to PBT-CCI and PBT-CCI-S sensors. However, each sensor type has its advantages. The PBT-CCI-CNT sensor boasts higher sensitivity but poorer linearity, making it more suitable for providing switch signals or pulse signals. Conversely, PBT-CCI-CNT-S, although not outstanding in sensitivity, exhibits excellent linearity across the entire range, making it more suitable for measuring strain. Considering the objective of this paper, which is to measure human joint movement information, the selection of the PBT-CCI-CNT-S sensor is deemed more appropriate.

### 3.3. Comprehensive Evaluation of the Electromechanical Performance of the PBT-CCI-CNT-S Strain Sensor

In this section, we detail the comprehensive evaluation of the PBT-CCI-CNT-S strain sensor’s electromechanical performance.

The upper detection limit of the PBT-CCI-CNT-S strain sensor is 60%. Multiple strain sensors of this type were prepared. While some exceptionally high-performing sensors can reach nearly 100%, it is worth noting that individual sensors experienced breakage beyond 60% strain. Consequently, we conservatively set 60% as the upper detection limit for this strain sensor. Regarding the lower detection limit, the PBT-CCI-CNT-S strain sensor achieves a detection limit of 0.9%, calculated based on three times the maximum noise (0.3% at a 3% cyclic stretch, which represents the minimum range of the rapid stretching device, as illustrated in Figure 3).

**Figure 6:**
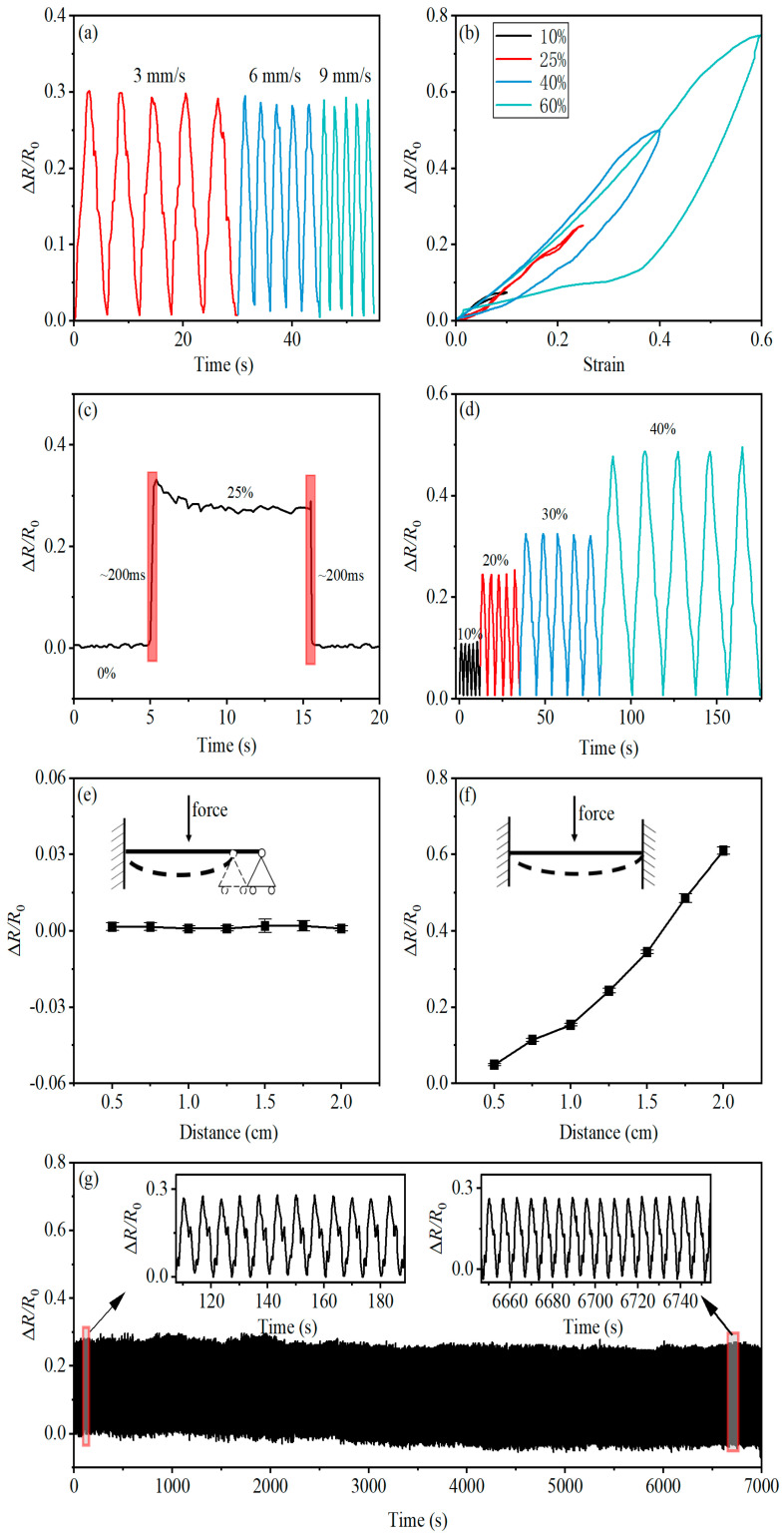
Mechanical and electrical performance testing of the PBT-CCI-CNT-S strain sensor. (**a**) The strain sensor is stretched to 25% strain at rates of 3 mm/s, 6 mm/s, and 9 mm/s, cycled five times to obtain the relative resistance-strain output characteristics. (**b**) Hysteresis performance of the strain sensor at 10%, 25%, 40%, and 60% strain. (**c**) Response characteristics of the strain sensor during stretching and releasing. (**d**) Relative resistance-strain output characteristics of the strain sensor cycled at 10%, 20%, 30%, and 40% strain. (**e**) Response of the strain sensor when one end is fixed and the other end is free during bending. (**f**) Response of the strain sensor when both ends are fixed during bending. (**g**) Durability performance testing of the strain sensor: stretched/released at a rate of 3 mm/s, stretched to 25% strain, cycled 1000 times.

Initially, we investigated the impact of stretching rates on the sensor’s output characteristics. Figure 6a illustrates the relative resistance variation of the PBT-CCI-CNT-S strain sensor at a 25% applied tensile strain under different stretching/release rates (3 mm/s, 6 mm/s, and 9 mm/s). The curves reveal minimal fluctuations in relative resistance, indicating an insignificant influence of stretching rates on the sensor’s output characteristics.

Subsequently, we examined the hysteresis performance of the strain sensor at various strain levels. The sensor underwent stretching to 10%, 25%, 40%, and 60% strain states at a rate of 0.6 mm/min, followed by gradual release at the same rate to capture the hysteresis characteristics (Figure 6b). Notably, the PBT-CCI-CNT-S strain sensor exhibited outstanding hysteresis at 10% and 25% strain states, with nearly overlapping stretching/release curves. Although a reduction in hysteresis occurred at 40% and 60% strain, the sensor consistently returned to its initial position.

We quantified the response speed of the PBT-CCI-CNT-S strain sensor, as depicted in Figure 6c. When stretched from 0% to 25% strain at a rate of 6 mm/s (equivalent to a sensor deformation of 1 mm), the mechanical structure stretching time was approximately 160 ms (1/6 s), and the response time in the strain-relative resistance data graph was around 200 ms. This rapid response, well within 50 ms, underscores the sensor’s suitability for capturing the swift movements of human joints. Similarly, the response time during the release phase was evaluated and also found to be within 50 ms.

Figure 6d illustrates the strain sensor’s stability at different strain levels (10%, 20%, 30%, and 40%). During stretching, the Δ*R*/*R*_0_ increased due to heightened sensor resistance, and upon strain release, Δ*R*/*R*_0_ promptly returned to zero, affirming the reliability of the sensor’s strain response mechanism across diverse strain conditions.

Given the sensor’s application in measuring human joint movements, often involving bent deformations, we conducted tests with one end fixed and the other end free (Figure 6e). The results indicated negligible changes in Δ*R*/*R*_0_. In the fixed-supported state at both ends (Figure 6f), Δ*R*/*R*_0_ increased gradually with the increment in downward distance.

Lastly, the durability of the PBT-CCI-CNT-S strain sensor was assessed. The sensor exhibited excellent output performance even after undergoing 1000 stretching/release cycles at a rate of 3 mm/s and a 25% strain stretching limit. Throughout the experiment, the resistance variation pattern remained remarkably stable, highlighting the sensor’s exceptional durability.

### 3.4. On-Trial Tests

Additionally, the strain sensor was applied to conduct practical measurements at various anatomical locations, including the wrist joint, elbow joint, finger joints, and leg joints.

**Figure 7:**
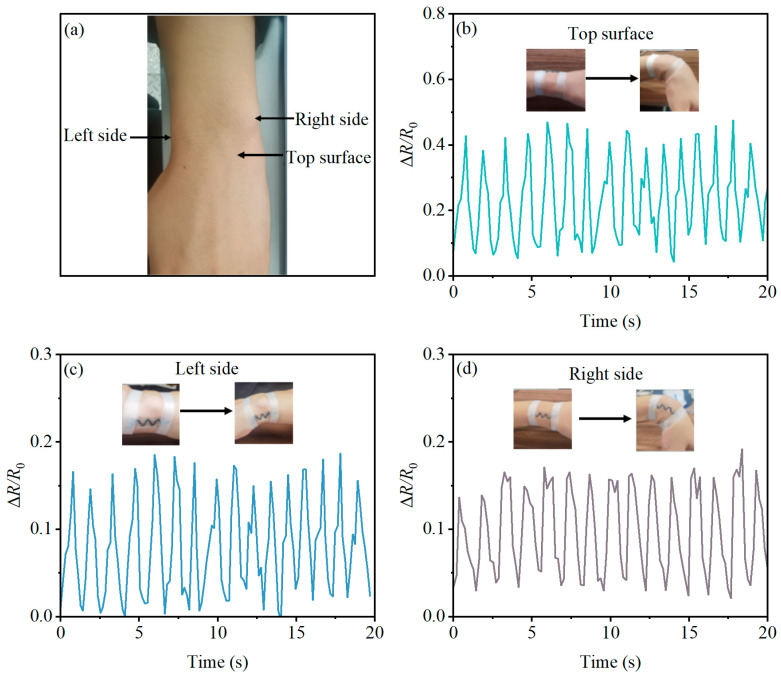
Monitoring wrist movement with the PBT-CCI-CNT-S strain sensor. (**a**) Schematic illustration of the strain sensor positioned on the wrist; (**b**) strain sensor response on the surface of the wrist during bending motion of the wrist joint; (**c**) strain sensor response on the left side of the wrist during bending motion of the wrist joint; (**d**) strain sensor response on the right side of the wrist during bending motion of the wrist joint.

The sensors were affixed to the upper, left, and right areas of the wrist joint, as illustrated in Figure 7a. The wrist joint was cyclically bent to approximately a 90° position, and the corresponding measurement data were systematically recorded. The resistivity variations, depicted in Figure 7b–d, illustrate the sensor’s response to the bending and relaxation movements of the wrist joint. During wrist flexion, where the wrist imparts tensile strain on the sensor, Δ*R*/*R*_0_ increases. Upon wrist extension, as the strain diminishes, the sensor’s resistance reverts to its initial baseline. Notably, the sensor not only accurately captures the frequency and velocity of wrist joint bending but also exhibits distinct strain patterns at different locations (upper, left, and right segments of the wrist joint) during flexion. Significantly, the upper part of the wrist joint’s skin experiences the greatest stretch during wrist bending, leading to a more pronounced stretching effect on the sensor. This observation aligns seamlessly with established principles in human movement physiology [24]. In terms of output response, the optimal position for monitoring wrist joint movement is found to be the upper part of the wrist.

**Figure 8:**
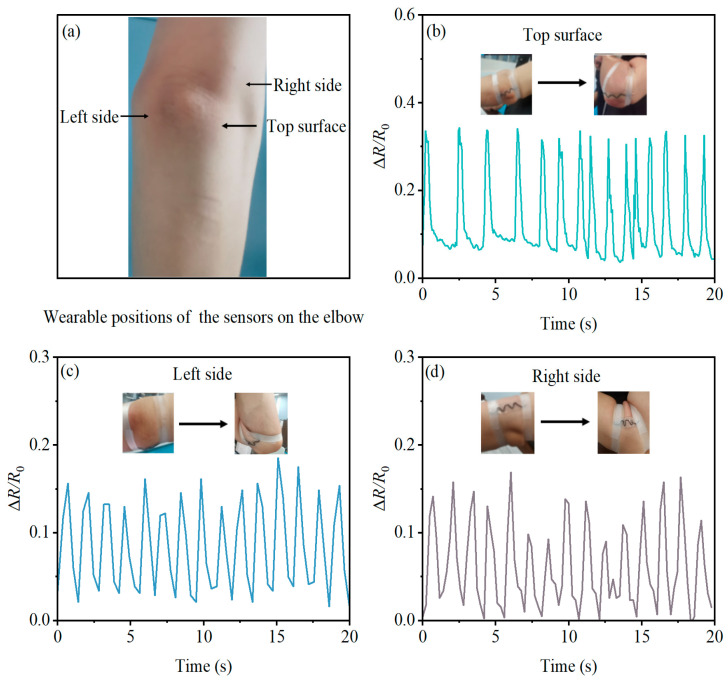
Monitoring elbow movement with the PBT-CCI-CNT-S strain sensor: (**a**) Schematic illustration of the strain sensor positioned on the elbow; (**b**) strain sensor response on the surface of the elbow during bending motion of the elbow joint; (**c**) strain sensor response on the left side of the elbow during bending motion of the elbow joint; (**d**) strain sensor response on the right side of the elbow during bending motion of the elbow joint.

Using the PBT-CCI-CNT-S for elbow joint measurements follows a similar procedure to that of the wrist joint. Illustrated in Figure 8a, the sensor is affixed to the outermost side, left side, and right side of the elbow joint. The elbow joint undergoes complete extension actions, and the sensor records output data at each position. As depicted in Figure 8b–d, the relative change in resistance (Δ*R*/*R*_0_) of the sensor increases with the degree of elbow joint flexion. Sensors at all three positions can accurately record the number and speed of elbow joint flexions, with the sensor at the outermost side of the elbow joint exhibiting the highest response in terms of the rate of resistance change. This observation indirectly suggests that, in relation to signal strength, the optimal location for sensor attachment is the outermost side of the elbow joint.

**Figure 9:**
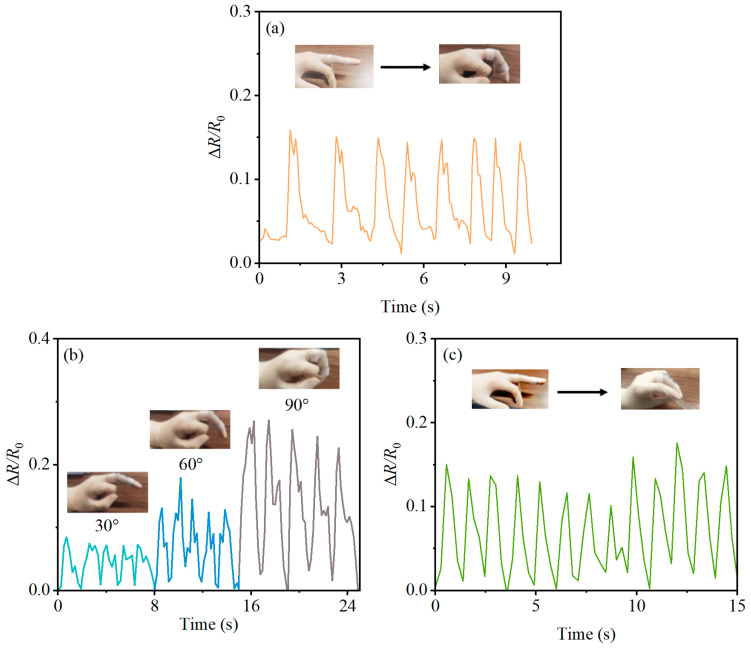
Detection of finger movement using the PBT-CCI-CNT-S strain sensor. (**a**) Response of the strain sensor to rapid finger movement (bent to 60°); (**b**) response of the strain sensor to finger movements at 30°, 60°, and 90° bends; (**c**) response of the strain sensor when rapidly picking up/putting down an object with the finger.

In light of the slender structure of fingers, effectively securing the sensor on both sides posed a challenge. Therefore, in this context, the PBT-CCI-CNT-S strain sensor was exclusively attached to the upper surface of the middle finger to evaluate its responsiveness to finger bending. As illustrated in Figure 9a, it was evident that the Δ*R*/*R*_0_ values of the strain sensor underwent real-time, periodic changes in response to the rapid bending and straightening of the finger. Varied degrees of finger bending elicited distinct resistance change responses, as portrayed in Figure 9b. With the bending angle increasing from 30° to 90°, the resistance of the sensor also escalated. This strain sensor not only recorded the frequency of finger bending but also quantified the extent of finger bending, as exemplified in Figure 9c, which illustrated the resistance change response when the finger was bent and straightened using a ruler.

**Figure 10:**
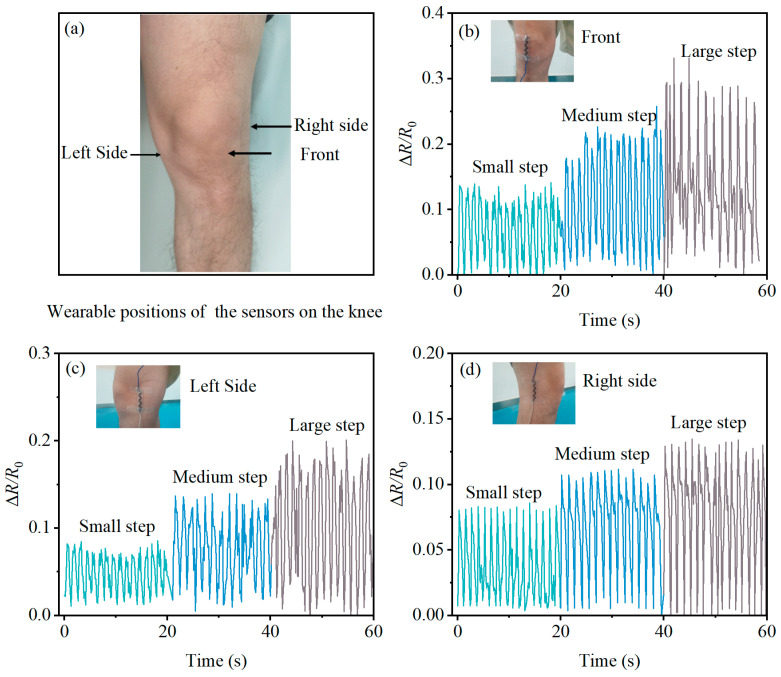
Detection of knee movement using the PBT-CCI-CNT-S strain sensor. (**a**) Schematic diagram of the strain sensor attached to the knee position; (**b**) response of the strain sensor attached to the front of the knee joint as the knee joint moves; (**c**) response of the strain sensor attached to the left side of the knee joint as the knee joint moves; (**d**) response of the strain sensor attached to the right side of the knee joint as the knee joint moves.

In the context of human walking or running, the PBT-CCI-CNT-S strain sensor proves effective in detecting knee joint movements. As illustrated in Figure 10a, the PBT-CCI-CNT-S strain sensor is affixed to the front, left side, and right side of the volunteer’s knee joint. The volunteer engages in walking, taking small steps (40 cm/step), medium steps (70 cm/step), and large steps (90 cm/step). The sensor’s response, represented in Figure 10b–d, exhibits periodic variations in the relative change in resistance (Δ*R*/*R*_0_) over time. This observation underscores the sensor’s capability to capture step frequency information during human locomotion.

Upon analyzing the sensor output signals for volunteers with different step lengths, it is evident that an increase in step length corresponds to a higher Δ*R*/*R*_0_ value. This suggests the sensor’s potential to discern information about the stride length during human movement. Furthermore, a comparison of resistance change values at different sensor positions reveals that attaching the sensor to the front of the knee joint yields the maximum peak signal.

## 4. Conclusions

This paper introduces a wearable sensor with a thread-embedded-in-PDMS structure designed for monitoring the motion of human wrist, elbow, finger, and knee joints. Initially, a sensor core was developed by applying conductive carbon ink (CCI) and carbon nanotubes (CNTs) onto a porous line made of polybutylene terephthalate (PBT). Subsequently, this core was encapsulated within a thin layer of polydimethylsiloxane (PDMS), resulting in the creation of a highly linear, flexible strain sensor capable of withstanding high strain levels. The study investigated the impact of introducing carbon nanotubes and modifying the shape of the conductive fabric on the wearable strain sensor. The findings indicated a substantial improvement in the sensitivity of the conductive fabric with the addition of carbon nanotubes. Moreover, the configuration of the core encapsulated in PDMS was observed to influence the output characteristics of the sensor. Sensors with a linearly encapsulated core in PDMS exhibited greater sensitivity but poorer linearity. Conversely, sensors with an S-shaped encapsulated core in PDMS showed relatively diminished sensitivity, yet demonstrated excellent linearity. The PBT-CCI-CNT-S sensor, selected for its superior linearity, was utilized to measure joint movements in the human body. A thorough evaluation of its performance, encompassing repetitive stretching/release capabilities, response characteristics at different strains, hysteresis, bending tests, and durability, was undertaken. Finally, the sensor was employed for in situ measurements of human joint movements (wrist, elbow, finger, and knee), further validating the applicability of the proposed strain sensor for monitoring human joint motions. The developed strain sensor is currently at the proof-of-concept prototype stage, with several challenges to address before reaching practical use or commercialization. One key concern is the impact of environmental temperature during wear, including low-temperature conditions, necessitating temperature compensation or calibration. Additionally, optimizing the power supply, for example, by harnessing kinetic energy from limb movements to generate electricity for the sensor, is a priority to enhance practicality and portability. We are committed to further refining the sensor to improve its practicality and move it closer to commercial viability.

## Figures and Tables

**Table 1:** Linear strain range and linearity of four types of strain sensors.

Sensor Type	Linear Range	Linearity in the Linear Range
PBT-CCI	0–10%	R^2^ = 0.9283
PBT-CCI-S	0–60%	R^2^ = 0.9849
PBT-CCI-CNT	0–10%	R^2^ = 0.9938
PBT-CCI-CNT-S	0–60%	R^2^ = 0.9991

## Data Availability

The experimental data are available from the authors.

## Supplementary Materials

The following supporting information can be downloaded at: https://www.mdpi.com/article/10.3390/mi14122250/s1, Figure S1. Schematic illustration of signal measurement and transmission for the strain sensor. Figure S2. Physical representation of hardware connections during strain sensor measurement.
