# Supplementary material for: Thread-Embedded-in-PDMS Wearable Strain Sensor for Real-Time Monitoring of Human Joint Motion
Source: Micromachines (Basel). 2023 Dec 17;14(12):2250. doi: 10.3390/mi14122250 (PMC10746070; doi:10.3390/mi14122250)
Supplement: Supplementary file 1 [file micromachines-14-02250-s001.zip › micromachines-2769940-supplementary.pdf]

micromachines

Thread-Embedded-in-PDMS Wearable Strain Sensor for Real-Time Monitoring of Human Joint Motion

Mingpeng Yang [1,2], Yongquan Liu [1,2], Wenjing Yang [3] and Jia Liu [1,2,*]

[1] School of Automation, Nanjing University of Information Science and Technology, 219 Ningliu Road, Nanjing 210044, China; mpyang@nuist.edu.cn (M.Y.); 202212490609@nuist.edu.cn (Y.L.)
[2] Jiangsu Collaborative Innovation Centre on Atmospheric Environment and Equipment Technology, Nanjing University of Information Science and Technology, 219 Ningliu Road, Nanjing 210044, China
[3] School of Atmospheric and Remote Sensing, Wuxi University, 333 Xishan Avenue, Wuxi 214105, China; ywj030114@outlook.com
* Correspondence: 001930@nuist.edu.cn; Tel.: +86-13951754608

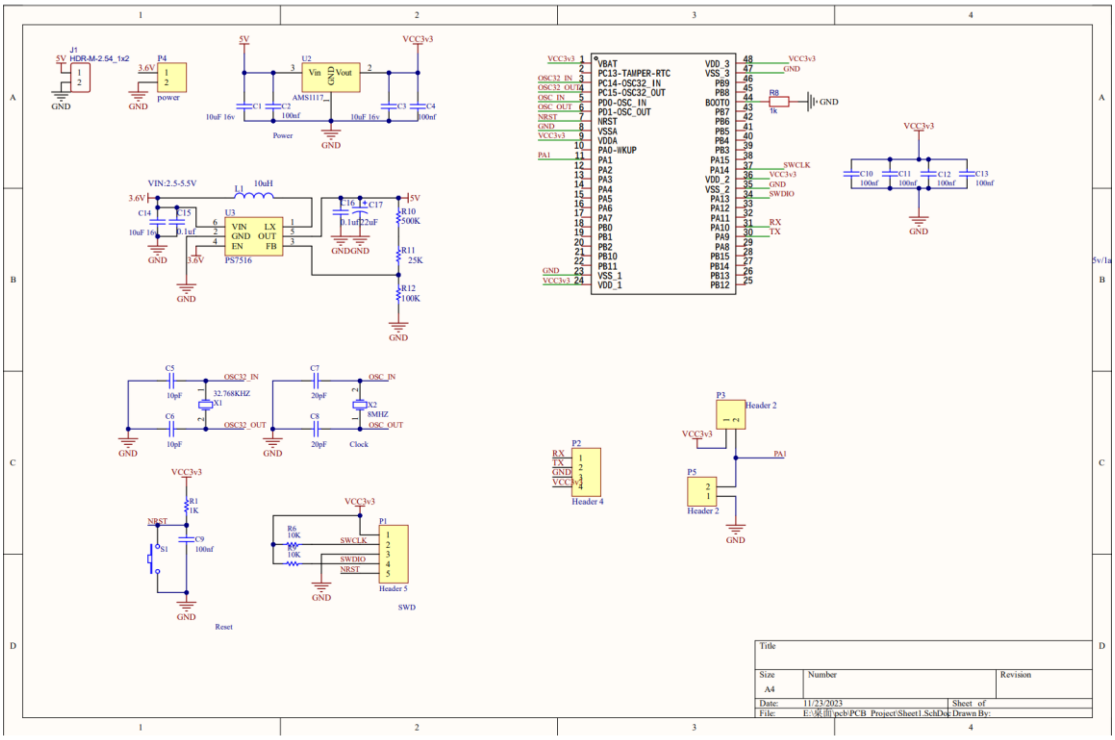

Figure S1. Schematic illustration of signal measurement and transmission for the strain sensor.

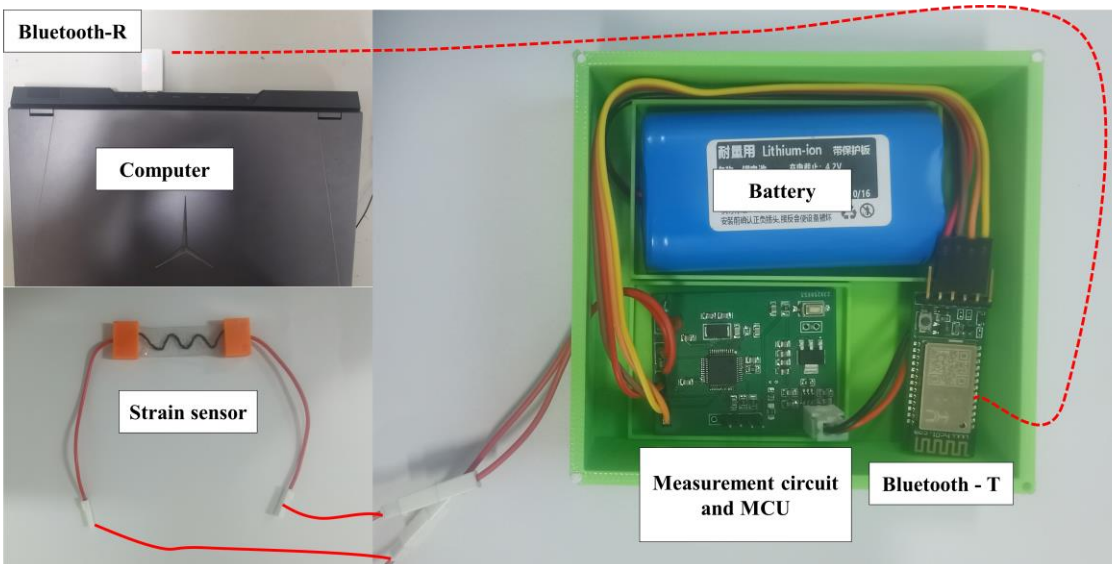

Figure S2. Physical representation of hardware connections during strain sensor measurement.
